# Genomic plasticity of the immune-related *Mhc *class I *B *region in macaque species

**DOI:** 10.1186/1471-2164-9-514

**Published:** 2008-10-30

**Authors:** Maxime Bonhomme, Gaby GM Doxiadis, Corrine MC Heijmans, Virginie Vervoort, Nel Otting, Ronald E Bontrop, Brigitte Crouau-Roy

**Affiliations:** 1 Université Paul Sabatier, Laboratoire Evolution et Diversité Biologique (EDB) UMR5174 UPS/CNRS, 118 Route de Narbonne, Toulouse 31062 cedex 9, France; 2 Biomedical Primate Research Centre, Department of Comparative Genetics and Refinement, Lange Kleiweg 139, 2288 GJ Rijswijk, The Netherlands

## Abstract

**Background:**

In sharp contrast to humans and great apes, the expanded *Mhc*-*B *region of rhesus and cynomolgus macaques is characterized by the presence of differential numbers and unique combinations of polymorphic class I *B *genes per haplotype. The MIB microsatellite is closely linked to the single class I *B *gene in human and in some great apes studied. The physical map of the *Mhc *of a heterozygous rhesus monkey provides unique material to analyze MIB and *Mamu*-*B *copy number variation and then allows one to decipher the compound evolutionary history of this region in primate species.

**Results:**

*In silico *research pinpointed 12 MIB copies (duplicons), most of which are associated with expressed *B*-genes that cluster in a separate clade in the phylogenetic tree. Generic primers tested on homozygous rhesus and pedigreed cynomolgus macaques allowed the identification of eight to eleven MIB copies per individual. The number of MIB copies present per haplotype varies from a minimum of three to six in cynomolgus macaques and from five to eight copies in rhesus macaques. Phylogenetic analyses highlight a strong transpecific sharing of MIB duplicons. Using the physical map, we observed that, similar to MIB duplicons, highly divergent *Mamu*-*B *genes can be present on the same haplotype. Haplotype variation as reflected by the copy number variation of class I *B *loci is best explained by recombination events, which are found to occur between MIBs and *Mamu*-*B*.

**Conclusion:**

The data suggest the existence of highly divergent MIB and *Mamu-B *lineages on a given haplotype, as well as variable MIB and *B *copy numbers and configurations, at least in rhesus macaque. Recombination seems to occur between MIB and *Mamu*-*B *loci, and the resulting haplotypic plasticity at the individual level may be a strategy to better cope with pathogens. Therefore, evolutionary inferences based on the multiplicated MIB loci but also other markers close to *B*-genes appear to be promising for the study of *B*-region organization and evolution in primates.

## Background

The major histocompatibility complex (*Mhc*) represents a multigene family that plays a crucial role in the generation of adaptive immune responses in vertebrate species. A key feature of the system is that most of its genes display abundant polymorphism at the population level. In addition, the number of *Mhc *class I or II genes may differ significantly between species as well as between individuals of a species [1]. *Mhc *polymorphisms have a profound impact on several features such as disease susceptibility, organ transplantation, and reproductive success [2-6]. In primates, considerable research has been conducted on the *Mhc *of rhesus (*Macaca mulatta*) and cynomolgus macaques (*Macaca fascicularis*), since these species are widely used as models for human diseases and biology. Simian immunodeficiency virus infection of macaques, for instance, is an important model for the study of AIDS [4,7]

The organization of the *Mhc *class I region of rhesus macaque – and probably most of the Old World Monkeys (OWM) – seems to be more complex than in humans and great apes. The *Mhc*-*A *and -*B *genes are shared between humans, great apes, and OWM, but OWM lack the *Mhc*-*C *gene, which arose by duplication in the Hominoid lineage [8]. OWM, however, possess many *Mhc*-*B *genes instead. In fact, *Mamu- *as well as *Mafa-A *and -*B *genes have been subjected to several rounds of duplication [9,10], as was confirmed recently by genomic sequencing [11-14]. Analysis of an expanded panel of rhesus macaques, originating from the Indian subcontinent as well as from China, revealed that the number and combination of *Mamu-A *and -*B *genes that are expressed per haplotype may differ extensively [15,16]. In addition, marked differences in expression levels were also observed for these class I genes. More recently, the study of *Mamu-A *and *Mafa-A *region configurations in Chinese rhesus macaques and pedigreed cynomolgus macaques, respectively, demonstrated that most *A *region configurations are old entities predating macaque speciation, whereas most allelic variation (> 95%) originated more recently [17]. Such results corroborate comparative studies illustrating that many *Mhc *loci and lineages predate speciation events but that the sharing of *Mhc *alleles between two primate species seems to be rare. Only a few cases of allele sharing have been documented [18,19]. In contrast, rhesus and cynomolgus macaques share a high number of *Mhc *class II alleles, as was determined by exon 2 *DRB *sequencing [20,21].

*Mhc *class I and class II gene families have been shown to evolve according to the birth-and-death process, rather than under concerted evolution [22-24]. In the birth-and-death process, new genes are created by repeated gene duplications, and some genes may later become pseudogenes or even be deleted from the genome. As a result, class I and II genes consist of a mixture of divergent genes, some of which have remained in the genome for a long period, together with a large number of closely related genes or pseudogenes. It appears that class I loci experience a much faster rate of birth-and-death evolution than do class II loci. Therefore, there seem to be no, or few, orthologous relationships of various class I loci among different mammalian suborders [25,26].

The class I *B *genes experienced a complex process of duplication during the evolution of macaques [11-15], which seems to have started 23–31 Mya ago [27]. Duplicated class I *B *genes, as well as other genes in the *Mhc *region, are exposed to selective pressures – mostly balancing selection – due to their role in antigen presentation, resulting in transpecific lineage sharing [28-30]. In addition, their haplotypic organization and their expression are likely the product of recombinational and mutational mechanisms promoted by these selective pressures. Nevertheless, relatively little is known about the haplotypic organization of duplicated class I *B *loci in macaques. In particular, it is important to have an insight into the number of class I *B *loci within a species and their distribution and position on haplotypes, as well as information about the level of gene expression and genetic divergence of *B *loci within haplotypes. These analyses are supported by the study of their proximate genomic environment using other genetic markers such as MIB.

In BAC clones from *Pan troglodytes*, *Gorilla gorilla*, and *Homo sapiens*, the microsatellite marker MIB (*D*6*S*2810) is physically close (~25 Kb) to the single class I *B *gene [31-33]. To enhance our knowledge of the organization and evolution of the class I *B *region in macaques, in this communication we further characterized the class I *B *region by studying MIB sequences (hereafter referred to as MIBs, MIB copies, or MIB loci), in addition to published *Mamu*-*B *gene sequences mapped onto haplotypes. First, we performed an *in silico *research of MIB loci by means of the published physical map of the rhesus macaque to identify their copy number, position, and association with *Mamu-B *genes and pseudogenes. Second, we designed generic primers in order to isolate MIB copies of selected rhesus and cynomogus macaque individuals and to describe their haplotypic distribution. We then investigated the phylogenetic relationships of (i) the identified MIB copies in these two species, and of (ii) the published *Mamu*-*B *sequences associated or not with MIB copies in the published material [11]. Our goal was to assess the genetic divergence of class I loci within species and within haplotypes, as well as their degree of orthology between species. In addition, we sought to determine whether duplicated MIB and *Mamu*-*B *loci are actually genetically linked, and to what extent patterns of linkage explain the haplotypic organization of the class I *B *region in macaques. We discuss the plastic organization of duplicated class I loci in the light of recombination and the birth-and-death process of evolution with gene duplication.

## Results

### In silico study of MIB and *B *loci on the rhesus macaque Mhc physical map

**Figure 1:**
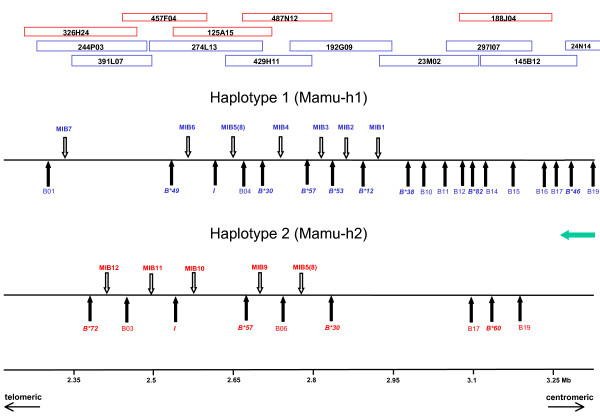
**Location of MIB copies and B genes on the physical map of rhesus macaque Mhc**. Blank and filled arrows indicate MIB and *Mamu*-*B *gene copies, respectively. BAC clones, from which MIBs and *Mamu*-*B *genes were retrieved, are positioned at the top of the figure. The names of the *Mamu*-B01 to -B19 genes – labelled as such by Daza-Vamenta and colleagues [11] and also annotated differently by Shiina and colleagues [42] – have been replaced by the latest *Mamu*-B loci/lineage names (B*) whenever possible; these represent "major'' or "minor'' expressed *Mamu*-B loci [15,16]. Green arrow indicates transcription direction.

The published physical map was used to identify *Mamu-B *genes and MIB copies *in silico *on both chromosomes (haplotypes 1 and 2) of the heterozygous animal studied [11]. On haplotype 1 (blue, Mamu-h1), the BAC clones analyzed covered the complete *Mhc *class I *B*, class III, and class II regions, while on haplotype 2 (red, Mamu-h2) the class I *B *region was only partially sequenced (Figure 1). In contrast to humans and great apes, the research determined 12 MIBs sequences located on the two rhesus haplotypes, all with the same orientation on the chromosomes (for exact location, see Additional file 1). Seven MIB copies, named MIB1 to MIB7, are located on haplotype 1, while five are on haplotype 2 (MIB5(8) to MIB12). Only one MIB copy is shared between the two haplotypes, and it was given the label MIB5(8). Nineteen *Mamu-B *genes were defined on the completely sequenced haplotype 1, of which the eight telomerically oriented *Mamu*-*B *loci are associated with one MIB copy each except for *Mamu*-B04 (Figure 1). The eleven other *B *genes, however, are not associated with MIB microsatellites. *Mamu-B *genes of haplotype 1, corresponding to serotype B11a, were shown to represent loci with different expression levels [15,16]. The names of the *Mamu*-B01 to -B19 genes of haplotype 1 and *Mamu*-B02 to -B7 and -B17 to -B19 genes of haplotype 2 – labelled as such by Daza-Vamenta and colleagues [11] – have been replaced by the latest *Mamu*-B loci/lineage names (B*) whenever possible [15,16]. These represent "major" or "minor" expressed *Mamu*-B loci [15,16]. Six out of the seven MIBs present on haplotype 1 are associated with expressed *Mamu*-*B *genes; the exception is MIB7 which is associated with the *Mamu*-B01 pseudogene. On haplotype 2, three out of the five MIBs present are associated with expressed *Mamu*-*B *genes.

### MIB analysis of the selected rhesus and cynomolgus macaques

**Figure 2:**
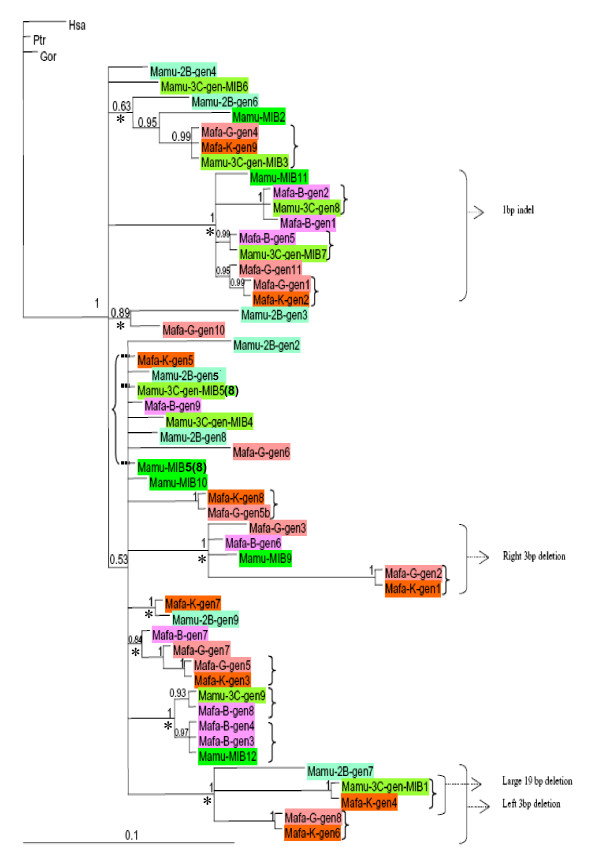
**Bayesian phylogenetic tree of flanking sequences of MIB copies in human, great apes, M. mulatta, and M. fascicularis**. Numbers at nodes are posterior probability values for node support. Braces identify strictly identical sequences differing only by the microsatellite repeat number, indicating an orthologous relationship between species, as well as allelic (microsatellite) polymorphism within a species and sometimes within the same individual. Brackets identify the shared indels. Stars pinpoint eight different well supported lineages. *M. mulatta *sequences are represented in green (light = individual 2B, semi-dark = 3C, dark = GenBank individual), *M. fascicularis *sequences are orange (individual K), pink (individual G) and purple (individual B). Hsa, Ptr, and Gor represent human, chimpanzee, and gorilla MIB sequences, respectively. Note that the two MIB5(8) copies show identical flanking sequences but a slightly different microsatellite repeat length.

To amplify MIB copies in selected macaques, generic primers have been designed by means of a highly conserved portion of the flanking sequences of MIB copies from the GenBank individual [11]. Based on subsequent cloning and sequencing, the Mhc homozygous rhesus macaques 3C, serotyped B11b, and 2B, serotyped B29, possess eight different MIBs each, and they share none. Monkey 3C has six MIB copies in common with the published haplotype 1 (MIB1 and MIB3 to 7; Figures 1 and 2).

In the three cynomolgus macaques, Bufo (B), Kraa (K), and Gayo (G), nine, nine, and eleven distinct *Mafa *MIB copies were isolated, respectively. The phylogenetic tree of all distinct MIB copies found in *M. mulatta*, *M. fascicularis*, *Homo sapiens*, *Pan troglodytes*, and *Gorilla gorilla*, excluding the microsatellite repeat array, is shown in Figure 2. Identical sequences within a species represent "alleles" of a copy in terms of repeat units, and identical – or nearly identical – sequences across species represent orthologous copies. Six MIB copies are identical between animals K and G, thus most probably being present on the shared haplotype b (see methods section). Therefore, the second haplotype of animals K and G must comprise the three and five other MIB copies, respectively. Interestingly, animal B with nine MIBs shares no MIB copy with K and G but contains two MIB duplicons – Mafa-B-gen3 and 4 – that seem to represent alleles of a given locus, because they show the same flanking sequence but a different microsatellite repeat length. However, thus far it is not known whether these MIBs are located on different haplotypes in *trans *orientation or *in cis *as replicons on the same chromosome. The number of MIB copies present per haplotype varies from a minimum of three to six in cynomolgus macaques and from five to eight copies in rhesus macaques.

In contrast, the two different macaque species studied show an extensive sharing of MIB copies (Figure 2). The haplotype of the homozygous animal 3C shares three MIB copies with cynomolgus macaque K (MIB1, MIB3, MIB5(8)), one with cynomolgus macaque B (MIB7) and one with cynomolgus macaque G (MIB3) (Figure 1, 2). Moreover, cynomolgus macaque B shares one MIB copy, MIB12, with the published rhesus macaque haplotype 2 (Figure 1, 2).

Mutations in the MIB sequence, excluding the microsatellite repeat array, consist mainly of substitutions and some indel events (for variable sites alignment, see Additional file 2). Table 1 shows the genetic diversity parameters (N, S, k, π) of MIB sequences (270 bp) found within individuals as well as within and between species. The average genetic divergence between MIB copies is nearly identical at the individual and species level. For example, the nucleotide diversity π = 0.034 to 0.056 in rhesus macaque individuals, while π = 0.036 in the overall rhesus species; π = 0.032 to 0.042 in cynomolgus individuals, while π = 0.037 in the overall species, and π = 0.035 in the rhesus/cynomolgus taxon. This suggests that most of the genetic divergence of MIB copies is captured at the individual level. Twenty-six out of 56 and 44 substitutions of MIB sequences of rhesus and cynomolgus macaques, respectively, segregate in both species; 30 substitutions segregate in rhesus, and only 18 in cynomolgus macaques.

**Table 1:** Genetic diversity parameters of MIB sequences, excluding the microsatellite repeat array

**individual/species**	N	S	k	π
*Mamu *(GenBank)	11	37	8.44	0.034
*Mamu*-3C	8	31	9.25	0.037
*Mamu*-2B	8	38	11.96	0.056
***Mamu*-(GenBank,2B,3C)**	**21**	**56**	**8.74**	**0.036**

*Mafa*-B	8	21	8.5	0.032
*Mafa*-K	9	32	9.64	0.040
*Mafa*-G	11	39	10.67	0.042
***Mafa*-(B, K, G)**	**22**	**44**	**8.80**	**0.037**

***Mafa*-*Mamu***	**36**	**69**	**8.26**	**0.035**

*Hominidae*	3	9	4.00	0.15

N = number of distinct MIB copies, S = number of segregating sites, k = average number of nucleotide differences, π = nucleotide diversity (i.e heterozygosity at the nucleotide level, [46]).

### Phylogenetic analysis of MIB and *Mamu-B *loci

The phylogenetic tree of MIBs of humans, great apes, and macaques depicts the high level of divergence of eight well-defined lineages of MIB copies in macaques, which are well supported by posterior probability values (PPV) (0.63 to 1) (Figure 2, marked by star). Rhesus and cynomolgus macaques share copies belonging to seven of these eight lineages, the exception being a lineage supported by a 0.84 PPV that is composed of MIB copies only present in *M. fascicularis*. This pattern of lineage sharing suggests that species-specific lineages are rare, and to be determined, more animals would need to be examined. Phylogenetic relationships are, however, not resolved for eleven distinct MIB copies (Figure 2, not marked by star). Among them, the closely related copies MIB5(8) and MIB10 – and related Mamu-2B-gen5, Mamu-2B-gen8, Mafa-B-gen9, and Mamu-3C-gen-MIB4 – show a slower evolutionary rate: namely, a shorter branch length in comparison to others.

**Figure 3:**
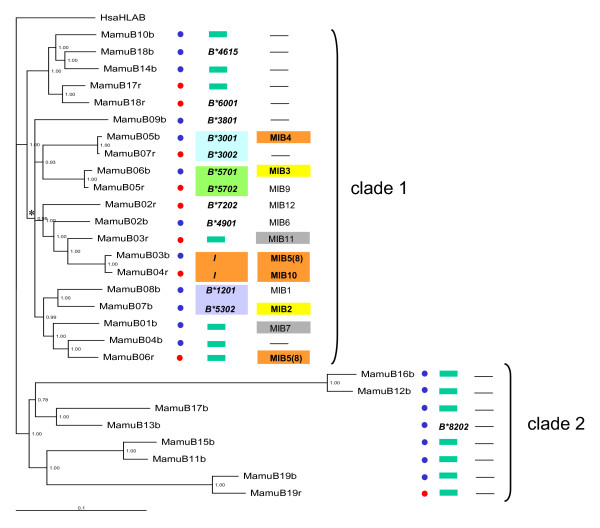
**Bayesian phylogenetic tree of Mamu-B gene (exons and introns) in human and M. mulatta**. Numbers at nodes are posterior probability values for node support. Blue and red circles indicate *Mamu*-*B *loci present on haplotype 1 and 2, respectively, of the published material [11]. Green rectangles indicate pseudogenes or low expressed genes. *Mamu*-*B *loci with a "B*" name are expressed genes. Next to *Mamu*-*B *loci are shown associated MIBs. *Mamu*-*B *loci with the same colour indicate phylogenetic relatedness, the same annotation was made for MIB loci. The figure do shows that only the locus *I *presents close linkage to MIB. The asterisk indicates the sub-clade (PPV = 0.95) within clade 1, which *B*-genes are associated with MIBs.

The phylogeny of *Mamu*-*B *loci present on haplotype 1 and 2 is shown in Figure 3, and adjacent MIB loci are superimposed. Two major clades of *Mamu*-*B *loci, each supported by a PPV of 1, diverged deep in the past. Clade 1 contains all but one expressed *B*-gene, while clade 2 is mainly composed of unexpressed genes or pseudogenes. All MIB copies are associated with *B*-genes of a sub-clade of clade 1 (Figure 3, asterisk). On the one hand, 73% (11/15) of *B*-genes of this sub-clade are expressed *B*-genes. On the other hand, 69% (11/16) of *Mamu*-*B *genes not associated with MIBs are pseudogenes. Additionally, *Mamu*-*B *genes of both haplotypes 1 and 2 belong to the different well-supported sub-clades (PPV from 0.93 to 1).

However, Figure 3 illustrates that any two closely related *Mamu*-*B *genes are not necessarily associated with two highly related MIB loci. In fact, the only phylogenetic congruence between *Mamu*-*B *and MIB occurs for the (non-classical) *Mamu*-*I *(*B*-like) gene, present on both haplotypes (Figure 3, highlighted in orange) and for the associated MIB5(8) and MIB10 sequences. In general, there seems to be no association between MIBs and *Mamu*-*B *(pseudo) genes along the class I *B *region.

## Discussion

### Highly plastic haplotypic organization of the class I B region in macaques

Phylogenetic analyses indicated that the ancestral MIB and *B*-linked loci started to duplicate in tandem in OWM after the OWM/Hominoid split, probably around 23–31 Mya ago [27]. Despite a tight linkage between MIB and the *B *gene in humans and some great apes, almost half the duplicated *B *genes are not linked to MIB loci in macaques. Moreover, these analyses pinpoint a deep split in the history of the duplications: the class I *B *region of macaques comprises a telomeric region where *B *genes are mostly expressed and associated with MIBs as well as a centromeric region where *B *genes are mostly pseudogenes and not associated with MIBs. A more precise historical duplication scheme would, however, require studying more genetic markers close to *B *genes.

The high number of MIB copies (from eight to eleven in homozygous and pedigreed individuals) and lineages (at least eight) in macaques indicate that several tandem duplication rounds occurred in the class I *B *region during OWM evolution, probably as an adaptive process. The number of MIB copies present per haplotype varies from a minimum of three to six in cynomolgus macaques and from five to eight copies in rhesus macaques, and may even be underestimated due to possible primer inconsistencies. The number of expressed *Mamu *and *Mafa*-*B *loci may vary accordingly. A high level of structural complexity has already been pointed out for the number and combination of transcribed *B *genes present per chromosome in rhesus macaques [15,16].

### High genetic diversity of duplicated class I loci

Phylogenetic and nucleotide diversity analyses show a high degree of orthology for the MIB copies present in both macaque species, which thus represents a transpecific sharing of MIB duplicons. This phenomenon is comparable to transpecific sharing of lineages and even alleles, which is frequently observed for *Mhc *loci of closely related species [28-30]. In addition, we observe that deeply divergent *Mamu*-*B *genes that coexist in the same species can also be present on the same haplotype, similar to MIB duplicons (Figure 3).

The variation of the average genetic divergence between any two MIB and *B*-gene copies may be directly related to the birth-and-death process that occurs with class I genes [22-24]. Here, we found that new MIB copies were created by repeated gene duplications, leading to clusters of similar copies, while other MIBs are old entities and therefore greatly divergent. The same seems to occur at *Mamu*-*B *loci, consistent with a mixture of divergent genes, some of which have remained in the genome for a long period, together with a large number of closely related genes or pseudogenes [22-24]. The birth-and-death process has been hypothesized to have a high turnover rate for class I *B *genes in mammals [25,26], leading to a lack of orthology when comparing distant species. However, a substantial level of orthology among rhesus and cynomolgus macaques was expected to be present and has thus been confirmed, since both species belong to the same genus.

### Recombination promotes plasticity and genetic diversity within class I haplotypes

The occurrence of recombination-like processes appears to be the most plausible explanation for the phylogenetic incongruence between *Mamu*-*B *and MIB loci. Recombination would explain the localization of a given *Mamu*-*B *locus next to a particular MIB on a given haplotype as well as its association with a different MIB on another haplotype. For instance, one allele of the B*57 locus is associated with MIB3 on haplotype 1 but with MIB9 on haplotype 2 (Figure 3). MIB3 and MIB9, however, are phylogenetically distant (Figure 2). Similarly, unequal crossing-over events may lead to the association of a given *Mamu*-*B *locus with a MIB on a given haplotype but with no MIB on another haplotype. For instance, while B*30 is associated with MIB4 on haplotype 1, it is not associated with a MIB on haplotype 2. More generally, frequent rearrangements by non-homologous recombination could explain the presence of differential numbers of *Mamu-B *and MIB loci across haplotypes (plasticity) but also of highly divergent *Mamu-B *and MIB loci on a given haplotype (genetic diversity within haplotype).

### Relationship between recombination and selective pressures occurring in the class I region

MIB duplicons with a slow evolutionary rate may shed light on a putative relationship between recombination and selective pressures occurring in the class I *B *region. For instance, the MIB5(8) and MIB10 copies, which are present on two divergent *Mhc *haplotypes in the rhesus macaque [11], are closely related and show very short branch lengths (Figure 2). Interestingly, MIB5(8) and MIB10 are associated with the non-classical *I *(*B*-like) locus on the two different haplotypes. According to the present data, this is the only *Mamu*-*B*/MIB combination that seems not to recombine. Although MIB5(8) and MIB10 are not coding sequences, their genetic linkage to the *I *locus may in principle allow the inference of evolutionary patterns involving this gene. In particular, genetic divergence of these MIB duplicons may be shaped by selective processes occurring at the *I *locus, via subsequent genetic hitch-hiking, which also slows the effect of recombination. The observations that some MIB duplicons, mostly associated with expressed *B *genes, are highly conserved would be in agreement with the preservation of a (ancestral) biological function by purifying selection directly on the coding gene or with the conservation of sequences involved in gene expression, in the vicinity of the coding gene. By contrast, positive (balancing) selection or relaxation of purifying selection (or both) may shape the diversification of duplicated copies [34-37] as part of the birth-and-death process, and may permit a reduction in the genetic linkage between loci by frequent recombination. Particularly in the *Mhc *region involved in host-pathogen interaction as part of immune defense reactions, recombination of class I loci may allow the build-up of new haplotypic combinations, resulting in a potential beneficial effect on the fitness of the organism with regard to pathogens.

## Conclusion

In conclusion, in addition to a high and uneven number of MIB and *B*-gene copies among *Mhc *haplotypes (plasticity), the data suggest the coexistence of highly divergent MIB and *B*-gene lineages on a given haplotype, in both rhesus and cynomolgus macaques. Such a high degree of plasticity and genetic diversity for *B *genes within haplotypes is the result of the diversification of the *Mhc *class I region, by the interaction of recombination with a birth-and-death (selective) process of evolution with gene duplication, probably as a strategy to better cope with pathogens. For comprehensive evolutionary inferences, future studies should investigate the constitution and genetic linkage for *B*-genes, MIBs, and other markers as well as the genomic environment of *B *genes on more haplotypes. In this way, a better insight into the complexity and the evolution of the *Mhc *class I *B *region in primates in relation to its biological function can be obtained.

## Methods

### In silico study of MIB on the rhesus macaque Mhc physical map

The 5' flanking sequence of human MIB clones obtained from previous studies [31-33] were blasted against BAC clones of the entire *Mhc *of one *M. mulatta *individual in GenBank [AC148659–AC148717] to obtain the different MIB copies of the published rhesus macaque *Mhc *[11].

### Selection of rhesus and cynomolgus macaques for MIB study

To define copy numbers and diversity of MIBs in macaque species, two consanguineous *Mhc *homozygous rhesus macaques were chosen for further analysis. Both animals had been thoroughly typed for their Mamu-A, -B, and -DR antigens by serotyping [38] as well as by molecular typing [15,39]. Monkey 2B is characterized by the B29 serotype that encodes at least two highly expressed *B *loci, B*44 and B*40, and most probably other *B *genes or pseudogenes with lower expression levels. The *B *region of the second rhesus macaque, 3C, is nearly identical to haplotype 1 of the *Mhc Mamu *published [11], encoding the serotype B11b that is characterized by three highly expressed *B *genes, B*12, B*30, and B*22, and by three *B *genes with lower expression levels, B*53, B*49, and B*70 [15,16]. Serotyping is not feasible in cynomolgus macaques, and molecular typing of the *Macaca fascicularis B *region (*Mafa-B*) has not yet been completed. However, molecular *Mafa-A *and *-DR *typing has been performed on a pedigreed cynomolgus family of four generations, and *Mhc *haplotypes could be determined by segregation analysis [17]. Therefore, three *Mhc *heterozygous animals of this family have been chosen, two of which, Kraa (K) and Gayo (G), share one *Mhc *haplotype (haplotype b), whereas the second haplotype differs. The third animal, Bufo (B), has no haplotype in common with the other two animals. These cynomolgus macaques are of Indonesian origin.

### Amplification of MIB copies in cynomolgus and rhesus macaques using generic primers

The generic primers MIBMamuF (5'-CCACTCTTCATACCACAGTCTC-3') and MIBMamuR (5'-ACCATGACCCCCTTCCCCAT-3') were designed in a conserved region identified on the alignment of the different rhesus macaque MIB sequences retrieved from GenBank, upstream and downstream of the previous human primer binding sites. PCR reactions were performed with 0.3 μM of each primer, and using the following cycling program: 94° for 5 min (denaturation step), 5 cycles at 94° for 1 min, 58° for 45 s, and 72° for 45 s, followed by 25 cycles of 94° for 45 s, 58° for 30 s, and 72° for 45 s. The final elongation step was extended to 30 min to generate a 3'dA overhang. PCR products were purified using the QIAGEN PCR purification Kit and cloned into the pDrive cloning vector using the QIAGEN cloning kit. Two independent PCRs were performed for each individual studied. After transformation using *E. coli *XLblue, 32 colonies were picked per PCR for plasmid isolation. The 64 isolated plasmids were sequenced using the BigDye terminator cycle sequencing kit. The samples were run on an automated capillary sequencing system (Applied Biosystems Genetic analyzer ABI3130*XL *and ABI3100) using the M13 forward primer. Sequences were analyzed using the seqman program (DNASTAR, Lasergene). Sequences were validated if detected at least twice in two independent PCR reactions. Distinct new MIB sequences (microsatellite repeat array and flanking sequence) were deposited in GenBank under the accession numbers FM177720–FM177743 for *M. fascicularis *and FM177744–FM177753 for *M. mulatta*. MIB sequences that were found to be identical in both *M. fascicularis *and *M. mulatta *have their own names and accession numbers. The correspondence between sequence names defined in the present study and accession numbers is shown in Additional file 3.

To obtain in humans and great apes the homologous sequence of the region amplified by the generic primers in macaques, we blasted the sequences obtained on *Pan troglodytes*, *Gorilla gorilla*, and *Homo sapiens *BAC clones (GenBank AB054536, CU104654, NT_113891).

### Data analysis

MIB sequences of *M. fascicularis *and *M. mulatta *were edited using the Sequencher≎ 4.7 software (Gene Codes Corporation) and aligned using MEGA3 [40]. Genetic diversity parameters were calculated using the software DnaSP 4.10 [41]. *Mamu*-*B *sequences were retrieved from Genbank ([11]s; see also accession number AB128049, from Shiina and colleagues [42]), and aligned using MEGA3 [40]. Phylogenetic analyses were conducted, based upon 270 bp of the MIB copies flanking sequence and upon 1080 bp of exonic and 1710 bp of intronic *Mamu*-*B *gene sequences, using a Bayesian phylogenetic analysis. The most likely substitution model was first inferred using a likelihood framework implemented in the software MODELTEST 3.7 [43]. This software tests 56 different substitution models and estimates the most likely one using the AIC criterium. The best model was HKY+G for MIB sequences, TIM+G for intronic sequences, and HKY+I+G, K81uf+I+G and TVM+I+G for the first, second, and third base of codons, respectively. Bayesian analyses were performed with MIB sequences and with a concatenation of exonic and intronic *Mamu*-*B *sequences, with their own substitution models, using the software MRBAYES[44], where two Markov Chains were run on 10 × 10^6 ^generations with a sampling each 100 generations. A run of this length allowed the standard deviation of allelic frequencies to pass below 0.01 and the potential scale reduction factor (PSRF) to reach a value of 1, as suggested by the authors. The first 25,000 trees (25%) were discarded from the analysis as a burn-in. The Bayesian phylogenetic analysis was subjected to indel coding to make the indel phylogenetically informative: indels of one base pair (bp) as well as more than one 1 bp were considered as a single character, and the different indels were coded as independent characters (or events). According to Saitou and Ueda [45], who showed that in primates the rate of nucleotide substitution was about 10 times higher than the rate of insertion and deletion for both nuclear and mitochondrial DNA, we weighted the indel events by a factor of 10 compared to the substitution events.

## Authors' contributions

The study was conceived by MB, GD, RB, and BCR, who also participated in its design.

BCR assisted with coordination as well. Sequence generation was carried out by VV, CH, NO, and MB, who also performed sequence and statistical analyses. MB and GD drafted the manuscript, which all authors have read and approved.

## Supplementary Material

Additional file 1 **Location of the MIB copies and B genes on the BAC clones of the rhesus macaque Mhc**[11]. This excel file indicates the different rhesus macaque BAC clones scanned for MIB copies and B genes, the location of the MIB copies and B genes on each BAC clone (in bp), and the distance between successive B genes and MIB copies (in bp). Click here for file

Additional file 2 **Alignment of the variable sites of the MIB flanking sequence.** Description: This pdf file shows the alignment and positions of the different variable sites of the MIB flanking sequence. These variable sites consist mainly in substitutions but indels can also occur at those sites. Other sites where only indels occur are not shown but the whole MIB sequences are available in Genbank (see Additional file 3). Click here for file

Additional file 3 **Correspondence between sequence names in the present study, and clones and accession numbers in the EMBL/GenBank/DDBJ databases. **This excel file indicates the sequence name, clone name and accession number for each new MIB sequence isolated in the present study. In addition, the name and accession number of each BAC clone of the heterozygous published rhesus macaque Mhc [11], from which MIB copies have been retrieved are also given. Note that the two accession numbers labelled with a star describe in fact the same MIB sequence (error when submitting the sequences). The microsatellite repeats number of each MIB sequence is also given. Click here for file
